# Surveying physical activity and nutritional status of pediatric leukemia patients

**DOI:** 10.3389/fnut.2025.1519399

**Published:** 2025-03-27

**Authors:** Ahlam Saleh Alhajri

**Affiliations:** Department of Food and Nutrition Sciences, College of Agricultural and Food Sciences, King Faisal University, Al-Ahsa, Saudi Arabia

**Keywords:** leukemia, cancer, physical activity, pediatric, diet, nutrition

## Abstract

**Background:**

Pediatric leukemia treatment often leads to challenges in maintaining adequate physical activity and nutritional status, both of which are crucial for overall health and recovery. Despite their importance, few studies have explored the interplay between physical activity, nutrition, and treatment stages in children undergoing leukemia treatment.

**Aim:**

This study aimed to evaluate the physical activity levels and nutritional status of pediatric leukemia patients, examining how these factors vary by gender, age, leukemia type, and treatment stage.

**Methods:**

A cross-sectional survey was conducted among 337 pediatric leukemia patients aged 6 to 12 years in Saudi Arabia. Data on physical activity and dietary habits were collected using an online questionnaire. Descriptive statistics, T-tests, and ANOVA were used to analyze the data.

**Results:**

The study found that 60% of participants failed to meet recommended physical activity levels. Males had significantly better nutrient-rich diets compared to females (Mean = 4.22 vs. 2.86, *p* < 0.0001). Children in the remission phase reported the lowest engagement in physical activity (Mean = 2.53, *p* < 0.0001), while those in the maintenance phase exhibited the highest energy levels (Mean = 4.45, *p* < 0.0001). Age differences were significant in motivation to participate in physical activities, with children aged 8–9 years reporting higher motivation (Mean = 2.97, *p* = 0.0249).

**Conclusion:**

The study highlights significant differences in physical activity and nutritional status based on gender, age, leukemia type, and treatment stage. Integrating personalized physical activity and nutritional interventions into pediatric leukemia care is essential for improving health outcomes and quality of life during treatment.

## Introduction

Leukemia is the most common pediatric cancer, accounting for approximately 30% of childhood malignancies worldwide (1). In 2020, 67,008 new cases of chronic leukemia (CL) were recorded globally, with a higher incidence in males (57.85%) compared to females (42.15%). The age-standardized incidence rate (ASIR) for CL was highest in North America (5.4), followed by Europe (5.2), Latin America (4.9), Oceania (4.6), Asia (4.0), and Africa (1.4) (2). That same year, CL-related deaths totaled 25,080, again with a higher mortality rate among males (58.86%) than females (41.44%).

Pediatric leukemia patients face significant health challenges due to the disease itself and its treatment, particularly regarding physical activity and nutrition. Leukemia treatments, including chemotherapy and radiation, often result in fatigue, muscle atrophy, and metabolic disturbances, negatively impacting physical activity levels (3, 4). Studies indicate that regular physical activity can mitigate these effects. For example, research shows that engaging in moderate physical activity during and after leukemia treatment leads to improved muscle strength, cardiovascular health, and overall physical function (5–8). A meta-analysis reported that structured exercise interventions improved fatigue levels by 25% and enhanced physical endurance by 20% in pediatric cancer patients. Additionally, exercise has been linked to improved mental health, with reductions in anxiety and depression symptoms by up to 30% in children undergoing leukemia treatment (9–12).

Despite these benefits, participation in physical activity among pediatric leukemia patients remains low, primarily due to treatment side effects, fatigue, and parental concerns about safety (13–16). The lack of standardized exercise guidelines tailored for this population further complicates the implementation of physical activity programs. Given the variability in individual health conditions and treatment stages, personalized exercise recommendations are necessary to optimize benefits without causing harm.

Nutritional challenges are another critical concern. Many pediatric leukemia patients experience malnutrition due to poor appetite, nausea, and gastrointestinal issues caused by chemotherapy (17–20). Both undernutrition and unhealthy weight gain have been reported, with studies indicating that up to 40% of pediatric leukemia patients suffer from malnutrition at some stage during treatment (21–23). Malnutrition has been linked to poorer treatment outcomes, including a 35% increased risk of infections, prolonged hospital stays, and reduced chemotherapy tolerance (24, 25). Nutritional interventions, such as individualized meal plans and supplementation, have shown promising results in preventing weight loss and managing nutrient deficiencies. One study demonstrated that targeted nutritional support reduced hospital readmission rates by 20% and improved treatment tolerance (26, 27). However, studies have shown that general dietary advice without personalization often fails to address the complex nutritional needs of these patients (21–23, 28–31).

Although physical activity and nutrition are critical for maintaining health during leukemia treatment, research often addresses these factors separately. However, muscle wasting and metabolic disturbances linked to treatment-related inactivity suggest an interplay between nutrition and exercise. A holistic approach that integrates both aspects could provide a more comprehensive framework for improving pediatric leukemia patients’ overall health and recovery. This study aims to assess the combined impact of physical activity and nutritional status in pediatric leukemia patients, identifying patterns, challenges, and opportunities for intervention. By bridging the gap between these two areas, the research seeks to provide evidence-based strategies for enhancing the quality of life and treatment outcomes in children undergoing leukemia therapy.

## Methods

### Study settings and participants

This study was conducted in pediatric oncology units across public hospitals in Saudi Arabia, specifically targeting children diagnosed with leukemia aged 6 to 12 years. The selected hospitals provide comprehensive care for pediatric cancer patients, including treatment and supportive services. The target population for this study consisted of children aged 6 to 12 years who have been diagnosed with leukemia and are currently receiving treatment, whether it be chemotherapy, radiotherapy, or a combination of therapies. To ensure a representative sample, participants were recruited from both inpatient and outpatient settings within the pediatric oncology units of the participating hospitals.

#### Inclusion criteria for the study

- Children aged 6 to 12 years.
- A confirmed diagnosis of leukemia (acute lymphoblastic leukemia, acute myeloid leukemia, or other specified types).
- Currently undergoing treatment for leukemia.
- Ability to understand and respond to survey questions, with the assistance of a parent or guardian.

#### Exclusion criteria

- Children with significant comorbidities or chronic illnesses that could impact physical activity levels or nutritional status, such as metabolic disorders or severe developmental disabilities.
- Children who are non-verbal or unable to provide informed consent, even with parental assistance.

### Recruitment process

Participants were recruited from pediatric oncology units within public hospitals in Saudi Arabia. The study targeted children aged 6–12 diagnosed with leukemia and currently undergoing treatment. This population was chosen to understand physical activity and nutritional needs in pediatric leukemia patients, aiming to guide healthcare improvements. Recruitment was done through:

- Hospital Records Review: Identifying eligible patients based on age and diagnosis.
- Direct Contact: Hospital staff approached families during clinic visits to explain the study, answer questions, and invite participation.
- Information Sessions: Conducted in oncology units to provide study details and its potential benefits.

This population was selected to reflect a range of treatment stages, leukemia types, and demographic backgrounds. This diversity allows for a comprehensive assessment of how different factors influence physical activity and nutrition in pediatric leukemia patients.

### Consent procedures

Informed consent was mandatory from all participants’ parents or guardians before enrollment. The consent process involved:

- Informative Overview: Providing parents with clear information on the study’s objectives, voluntary nature, and confidentiality measures.
- Child Assent: When appropriate, children were also informed about the study in age-appropriate terms and asked for their assent.
- Emphasis on Voluntary Participation: Families were assured of their right to withdraw at any time, with no impact on medical care.
- Confidentiality Assurance: Data anonymization was emphasized, ensuring responses would not be linked to identifiable information.

These procedures helped ensure informed and voluntary participation, respecting ethical considerations for research involving minors and vulnerable populations.

An online survey instrument was utilized to collect data, allowing for broad participation while ensuring convenience for both the children and their caregivers. The survey was designed to be user-friendly, incorporating visual aids and simplified language to accommodate the developmental level of the target age group. Parents or guardians were required to provide consent prior to the children’s participation in the survey, and they assisted their children in completing the online questionnaire when necessary.

### Sampling

A stratified random sampling method (32) was employed to ensure a representative sample of pediatric leukemia patients aged 6 to 12 years. Participants were stratified based on key variables, including hospital affiliation, gender, age group (sub-categorized into 6–7 years, 8–9 years, and 10–12 years), and type of leukemia (e.g., acute lymphoblastic leukemia, acute myeloid leukemia) to evaluate potential differences in physical activity levels and nutritional status. The target sample size was calculated using Cochran’s formula (33), aiming for a minimum of 300 to achieve sufficient statistical power for analysis. Eligible participants were identified through medical records at participating hospitals, and families were approached during routine clinic visits or hospital admissions, with parents or guardians invited to consent to their child’s participation in the online survey. To enhance participation rates, outreach efforts included information sessions in pediatric oncology units to explain the study’s purpose and follow-up communications to remind families about the survey.

### Questionnaire design

The survey aims to gather comprehensive data on the physical activity levels and nutritional intake of children diagnosed with leukemia. The questionnaire consists of multiple sections that cover demographics, physical activity patterns, dietary habits, potential correlations between physical activity and nutrition, and barriers to maintaining an active lifestyle and healthy diet during treatment.

Section 1 collects demographic information about the child, including age, gender, duration since diagnosis, treatment phase, and type of leukemia, which are crucial for understanding the background of the participants and analyzing how these factors may influence their physical activity and nutritional status. Section 2 focuses on physical activity patterns, asking caregivers about the frequency of their child’s engagement in various physical activities, energy levels, participation in structured exercise, and perceived motivation. This section uses a Likert scale to quantify responses, allowing for nuanced insights into the children’s activity levels and any challenges they may face. Section 3 evaluates nutritional intake and dietary habits, asking caregivers about their child’s eating patterns, diet quality, consumption of high-sugar or high-fat foods, and challenges related to treatment side effects. This section also employs a Likert scale to gauge the frequency of these dietary behaviors. Section 4 explores the correlations between physical activity and nutritional status, investigating how physical activity affects appetite, the relationship between physical health and dietary balance, and how energy levels may fluctuate based on nutrition. Section 5 identifies barriers to physical activity and nutrition, including treatment-related fatigue, side effects, family or environmental factors, and emotional challenges. This section provides insight into the difficulties families encounter in supporting their child’s health during treatment. The survey tool utilized Likert-scale questions with response categories designed to have regular intervals, allowing for meaningful comparisons and statistical calculations. The scale used in most sections followed a five-point structure (e.g., Never–Rarely–Sometimes–Often–Always or Very Poor–Poor–Average–Good–Excellent), which was treated as an ordinal variable but assumed to approximate interval-level data based on prior studies using similar methodologies.

To ensure clarity and accuracy in responses, the questionnaire was carefully designed with caregiver comprehension in mind. For subjective measures such as energy levels and motivation, caregivers were provided with contextual examples and guided descriptors within the survey to help them assess and classify their child’s experiences on the Likert scale. For instance, ‘structured exercise’ was explicitly defined with examples such as physical therapy or sports to facilitate a shared understanding. Similarly, energy levels were assessed based on observable daily activity patterns rather than requiring caregivers to make clinical calculations.

Regarding dietary habits, caregivers were asked about general patterns rather than requiring precise nutritional calculations. The questionnaire inquired about the frequency of consuming nutrient-rich foods (e.g., fruits, vegetables, whole grains) and high-sugar or high-fat foods (e.g., sweets, fast food), using layman’s terms rather than complex nutritional metrics. This approach ensured accessibility while capturing essential dietary trends.

For sections investigating the impact of physical activity on nutrition and energy fluctuations, questions were framed based on observable behaviors. Caregivers were asked to assess changes in appetite following physical activity and their perceptions of how overall diet quality influenced energy levels. Similarly, barriers such as treatment-related fatigue and emotional challenges were examined using caregiver observations of their child’s struggles rather than requiring medical expertise.

While several validated tools, such as STRONGkids, SGNA, and STAMP, are commonly used to assess pediatric nutritional status, this study employed a tailored questionnaire to capture leukemia-specific dietary considerations. Existing tools primarily focus on general pediatric populations or malnutrition risk screening, whereas our instrument was designed to assess the interplay between physical activity and nutrition in pediatric leukemia patients within the Saudi Arabian healthcare context. The questionnaire incorporated elements from validated tools while expanding its scope to include chemotherapy-related appetite changes, treatment-induced metabolic alterations, and dietary intake patterns. This approach allowed for a more comprehensive, leukemia-specific assessment while maintaining feasibility within an online survey format.

A certified translator (34) translated the questionnaire from English to Arabic. The accuracy of the translated version was subsequently validated by two professors from the eHealth department at King Faisal University. They proposed several grammatical adjustments, which were incorporated into the Arabic version of the questionnaire. To further refine the instrument, a pilot study was conducted with a sample of 11 parents of leukemia affected children. The data collected from this exploratory study were analyzed, and the Cronbach’s alpha coefficient for all items was calculated. The coefficient exceeded 0.7, indicating robust internal consistency and reliability of the questionnaire (35).

To ensure construct validity, the questionnaire was developed based on an extensive literature review of validated instruments used in pediatric oncology and nutrition research. The dimensions assessed (e.g., physical activity frequency, energy levels, and meal patterns) were aligned with established concepts in pediatric health and nutritional science. Additionally, expert validation was conducted, where specialists in pediatric oncology, nutrition, and survey methodology reviewed the tool to confirm that the questions appropriately captured the intended constructs.

For criterion validity, the tool was pilot-tested with a sample of parents of pediatric leukemia patients (n = 11) to assess clarity, reliability, and relevance. The internal consistency of the tool was evaluated using Cronbach’s alpha, which exceeded 0.7, indicating acceptable reliability. While direct validation against clinical measures (e.g., biochemical markers) was not conducted due to the survey-based nature of the study, the tool was designed to be comparable to similar validated instruments in pediatric health research.

### Data collection

Data for this study were collected over 4 weeks using a structured survey questionnaire, available in both English and Arabic. The questionnaire was distributed exclusively through online platforms to ensure broad accessibility and convenience for participants across various regions in Saudi Arabia, including urban, suburban, and rural areas. The online distribution leveraged social media, hospital networks, and community forums to reach a diverse population. This digital approach facilitated the collection of comprehensive data ensuring participants could complete the survey at their convenience. At the end of 4 weeks, 352 responses were received, out of which 15 responses were incomplete. After removing incomplete responses, a final sample of 337 was considered for data analysis.

### Data analysis

To achieve the study’s objectives, the data were analyzed using the Statistical Package for the Social Sciences (SPSS, IBM Version 24). Descriptive statistics, including means and standard deviations, were employed to present the demographic characteristics of the participants. Given the relatively large sample size, we used the mean as the primary measure of central tendency, which is a common approach when Likert scales are structured with evenly spaced response options. Additionally, the standard deviation was included to reflect variability in responses, and in cases where response distributions were skewed, the median was considered for supplementary analysis. We recognize that for strictly ordinal data, the median is often preferred; however, in this study, the decision to use the mean was based on the practical interpretability of results, comparability with similar research, and the fact that Likert responses were assumed to be equidistant. Additionally, a two-sample t-test with unequal variances and a one-way ANOVA were conducted to analyze the data further.

### Ethical considerations

This study adhered to strict ethical guidelines to ensure the safety and well-being of participants, particularly given the vulnerable nature of pediatric leukemia patients. Ethical approval was obtained from the relevant institutional review board at King Faisal University (KFU-REC-2024-OCT-ETHICS2707). Informed consent was required from parents or guardians before participation, providing detailed information about the study’s purpose, procedures, potential risks, and benefits. Assent from the children was sought where appropriate. Participant confidentiality was ensured by anonymizing all data collected through the online survey, and it was made clear that participation was voluntary, with the option to withdraw at any time without affecting medical care. Measures were also in place to manage any distress caused by survey questions, allowing participants to skip uncomfortable items.

## Results

Furthermore, the mean value of 3.21 for the question regarding how often the child experiences difficulty eating due to nausea, loss of appetite, or other treatment-related symptoms indicates that, on average, participants report experiencing these difficulties “Sometimes.” Findings also indicated positive perceptions regarding the interplay between physical activity, overall health, and nutrition among pediatric leukemia patients. The mean value of 4.04 for the impact of physical activity on appetite suggests that caregivers believe increased physical activity strongly stimulates hunger and encourages healthier eating behaviors in their children. Similarly, the mean value of 4.07 reflects a strong perception that the child’s overall physical health is closely linked to their ability to maintain a balanced diet, highlighting the importance of good health in facilitating proper nutrition. Lastly, a mean of 4.05 indicates that caregivers feel the child’s energy levels for physical activities significantly vary based on their nutrition, reinforcing the idea that dietary intake directly influences physical performance and overall well-being in children undergoing treatment (Tables 1, 2).

**Table 1:** Participants demographics.

	Variables	*N*	Relative frequency
Gender	Male	187	55.5%
	Female	150	44.5%
Leukemia type	Acute Lymphoblastic Leukemia (ALL)	33	9.8%
	Acute Myeloid Leukemia (AML)	90	26.7%
	Chronic Lymphocytic Leukemia (CLL)	42	12.5%
	Chronic Myeloid Leukemia (CML)	5	1.5%
	Juvenile Myelomonocytic Leukemia (JMML)	61	18.1%
	Mixed Phenotype Acute Leukemia (MPAL)	106	31.5%
Age (in years)	6–7	73	21.7%
	8–9	99	29.4%
	10–12	165	49.0%
Treatment Stage	Initial treatment	68	20.2%
	Maintenance phase	136	40.4%
	Relapse	69	20.5%
	Remission	64	19.0%

**Table 2:** Participants physical activity and nutritional (diet) related factors.

Factors		Mean	Standard Deviation
Physical Activities	Engagement in physical activities such as walking, running, or playing	3.63	1.44
	Child’s energy levels	3.77	1.29
	Engagement in structured exercise (e.g., physical therapy, sports)	2.74	1.45
	Motivation to participate in physical activities	2.62	1.51
Dietary habits	Child consumption of meals according to a regular eating schedule	3.49	1.45
	Quality of the child’s diet in terms of nutrient-rich foods	3.61	1.37
	Frequency of consuming high-sugar or high-fat foods	3.87	1.14

The analysis of the T-test results in Table 3 indicates that gender influences certain physical activity and nutritional factors among participants. Males reported slightly higher engagement in physical activities (Mean = 3.70) and energy levels (Mean = 3.86) than females (Means = 3.55 and 3.67, respectively), but these differences were not statistically significant (*p* = 0.3343 and *p* = 0.1704). Males also had lower engagement in structured exercise (Mean = 2.66) compared to females (Mean = 2.85), with a non-significant *p*-value of 0.2358. Notably, males exhibited significantly better diet quality in terms of nutrient-rich foods (Mean = 4.22) compared to females (Mean = 2.86), with a significant difference (*p* < 0.0001). Other factors, like meal consumption regularity and high-sugar food frequency, showed no significant gender differences. Overall, only the quality of the child’s diet revealed a significant difference based on gender.

**Table 3:** T-test results assessing difference between participants physical activity and nutritional (diet) related factors—based on their gender.

Factors	Gender	N	Mean	Variance	*p*-value
Engagement in physical activities such as walking, running, or playing	Male	187	3.70	2.09	0.3343
	Female	150	3.55	2.13	
Child’s energy levels	Male	187	3.86	1.71	0.1704
	Female	150	3.67	1.61	
Engagement in structured exercise (e.g., physical therapy, sports)	Male	187	2.66	2.20	0.2358
	Female	150	2.85	1.98	
Motivation to participate in physical activities	Male	187	2.50	2.32	0.0923
	Female	150	2.78	2.16	
Child consumption of meals according to a regular eating schedule	Male	187	3.60	1.95	0.1452
	Female	150	3.37	2.30	
Quality of the child’s diet in terms of nutrient-rich foods	Male	187	4.22	1.06	<0.0001^*^
	Female	150	2.86	1.92	
Frequency of consuming high-sugar or high-fat foods	Male	187	3.88	1.16	0.8174
	Female	150	3.85	1.51	

* Statistically significant difference at *p* < 0.05 confidence interval.

The ANOVA results in Table 4 reveal significant differences in physical activity and nutritional factors among participants based on the type of leukemia. Engagement in physical activities showed a significant variance (*p* = 0.007), with an overall mean of 4.18, indicating that children with different leukemia types engage differently in physical activities. The energy levels of the children were notably higher overall (Mean = 4.15) with a significant *p*-value (< 0.0001), suggesting a variation in energy levels across leukemia types. Structured exercise engagement also differed significantly (*p* = 0.0356), with an overall mean of 3.30. However, motivation to participate in physical activities did not show significant differences (*p* = 0.3062). Regular meal consumption significantly varied (*p* < 0.0001), with a mean of 3.91, indicating differences in eating habits. The quality of the child’s diet in terms of nutrient-rich foods also displayed a significant difference (*p* = 0.0428), with an overall mean of 3.55. Lastly, the frequency of consuming high-sugar or high-fat foods showed no significant differences (*p* = 0.9704). Overall, the type of leukemia impacts several aspects of physical activity and nutritional habits significantly.

**Table 4:** ANOVA results assessing difference between participants physical activity and nutritional (diet) related factors—based on type of Lukemia.

Factors	Type of Leukemia	*N*	Mean	Variance	*p*-value
Engagement in physical activities such as walking, running, or playing	ALL	33	4.18	1.09	0.007^*^
	AML	90	3.61	2.35	
	CLL	42	4.00	1.51	
	CML	5	4.00	2.00	
	JMML	61	3.75	1.69	
	MPAL	106	3.25	2.47	
Child’s energy levels	ALL	33	4.15	1.20	<0.0001^*^
	AML	90	3.68	1.82	
	CLL	42	4.38	0.92	
	CML	5	4.60	0.30	
	JMML	61	4.07	1.33	
	MPAL	106	3.29	1.79	
Engagement in structured exercise (e.g., physical therapy, sports)	ALL	33	3.30	2.09	0.0356^*^
	AML	90	2.68	2.36	
	CLL	42	2.36	1.84	
	CML	5	3.60	1.80	
	JMML	61	2.52	1.79	
	MPAL	106	2.86	2.07	
Motivation to participate in physical activities	ALL	33	3.21	2.73	0.3062
	AML	90	2.56	2.16	
	CLL	42	2.69	2.46	
	CML	5	2.60	3.30	
	JMML	61	2.49	2.22	
	MPAL	106	2.56	2.08	
Child consumption of meals according to a regular eating schedule	ALL	33	3.91	1.46	<0.0001^*^
	AML	90	3.29	2.19	
	CLL	42	4.05	1.02	
	CML	5	4.00	1.00	
	JMML	61	4.13	1.02	
	MPAL	106	2.93	2.67	
Quality of the child’s diet in terms of nutrient-rich foods	ALL	33	3.55	2.13	0.0428^*^
	AML	90	3.41	1.98	
	CLL	42	4.21	1.20	
	CML	5	4.20	1.70	
	JMML	61	3.49	1.79	
	MPAL	106	3.61	1.99	
Frequency of consuming high-sugar or high-fat foods	ALL	33	3.94	1.50	0.9704
	AML	90	3.84	1.21	
	CLL	42	3.74	1.66	
	CML	5	3.80	1.70	
	JMML	61	3.92	1.08	
	MPAL	106	3.90	1.37	

* Statistically significant difference at *p* < 0.05 confidence interval.

The ANOVA results in Table 5 indicate differences in physical activity and nutritional factors based on the age of participants. Engagement in physical activities, with means of 3.52 for ages 6–7, 3.58 for 8–9, and 3.72 for 10–12, showed no significant differences (*p* = 0.5726). Similarly, children’s energy levels did not vary significantly across age groups (*p* = 0.3455), with means of 3.75, 3.93, and 3.69, respectively. However, motivation to participate in physical activities exhibited a statistically significant difference (*p* = 0.0249), suggesting that older children may feel more motivated compared to younger ones, with means of 2.47, 2.97, and 2.49. Child consumption of meals according to a regular eating schedule did not reveal significant differences (*p* = 0.1413), with means of 3.32, 3.73, and 3.44. The quality of the child’s diet regarding nutrient-rich foods also showed no significant variance (*p* = 0.6075), while the frequency of consuming high-sugar or high-fat foods remained consistent across ages (*p* = 0.2642). Overall, while most factors remained stable across age groups, motivation to engage in physical activities varied significantly.

**Table 5:** ANOVA results assessing difference between participants physical activity and nutritional (diet) related factors—based on age.

Factors	Age (in years)	*N*	Mean	Variance	*p*-value
Engagement in physical activities such as walking, running, or playing	6–7	73	3.52	2.20	0.5726
	8–9	99	3.58	1.88	
	10–12	165	3.72	2.22	
Child’s energy levels	6–7	73	3.75	1.77	0.3455
	8–9	99	3.93	1.49	
	10–12	165	3.69	1.73	
Engagement in structured exercise (e.g., physical therapy, sports)	6–7	73	2.63	1.90	0.2681
	8–9	99	2.94	1.98	
	10–12	165	2.67	2.27	
Motivation to participate in physical activities	6–7	73	2.47	2.03	0.0249^*^
	8-9	99	2.97	2.25	
	10–12	165	2.49	2.29	
Child consumption of meals according to a regular eating schedule	6–7	73	3.32	2.16	0.1413
	8–9	99	3.73	1.67	
	10–12	165	3.44	2.33	
Quality of the child’s diet in terms of nutrient-rich foods	6–7	73	3.73	1.98	0.6075
	8–9	99	3.52	1.80	
	10–12	165	3.62	1.93	
Frequency of consuming high-sugar or high-fat foods	6–7	73	3.85	1.16	0.2642
	8–9	99	3.73	1.65	
	10–12	165	3.96	1.17	

* Statistically significant difference at *p* < 0.05 confidence interval.

The ANOVA results presented in Table 6 reveal significant differences in physical activity and nutritional factors based on the treatment stage of participants. Engagement in physical activities shows a notable variation, with means of 3.84 during initial treatment, 3.99 in the maintenance phase, 3.75 during relapse, and a significantly lower mean of 2.53 in remission (*p* < 0.0001). Similarly, children’s energy levels also differ significantly across treatment stages, with means of 3.81, 4.45, 3.01, and 3.13, respectively (*p* < 0.0001). Motivation to participate in physical activities exhibits a significant decline from initial treatment (3.21) to remission (2.38) (*p* < 0.0001). Furthermore, child consumption of meals according to a regular eating schedule is significantly higher during initial treatment (4.13) compared to remission (2.44) (*p* < 0.0001). In contrast, factors like the quality of the child’s diet in terms of nutrient-rich foods and frequency of high-sugar or high-fat food consumption did not show significant differences across treatment stages (*p* = 0.3852 and *p* = 0.3631, respectively).

**Table 6:** ANOVA results assessing difference between participants physical activity and nutritional (diet) related factors—based on treatment stage.

Factors	Variables	*N*	Mean	Variance	*p*-value
Engagement in physical activities such as walking, running, or playing	Initial treatment	68	3.84	1.69	<0.0001^*^
	Maintenance phase	136	3.99	1.41	
	Relapse	69	3.75	2.22	
	Remission	64	2.53	2.48	
Child’s energy levels	Initial treatment	68	3.81	1.56	<0.0001^*^
	Maintenance phase	136	4.45	0.75	
	Relapse	69	3.01	1.63	
	Remission	64	3.13	1.83	
Engagement in structured exercise (e.g., physical therapy, sports)	Initial treatment	68	3.12	2.19	0.1026
	Maintenance phase	136	2.70	2.12	
	Relapse	69	2.55	1.93	
	Remission	64	2.64	2.08	
Motivation to participate in physical activities	Initial treatment	68	3.21	2.43	<0.0001^*^
	Maintenance phase	136	2.76	2.58	
	Relapse	69	2.01	1.25	
	Remission	64	2.38	1.70	
Child consumption of meals according to a regular eating schedule	Initial treatment	68	4.13	1.25	<0.0001^*^
	Maintenance phase	136	3.99	1.21	
	Relapse	69	2.87	2.88	
	Remission	64	2.44	1.71	
Quality of the child’s diet in terms of nutrient-rich foods	Initial treatment	68	3.51	1.60	0.3852
	Maintenance phase	136	3.55	2.03	
	Relapse	69	3.87	1.64	
	Remission	64	3.58	2.22	
Frequency of consuming high-sugar or high-fat foods	Initial treatment	68	3.68	1.06	0.3631
	Maintenance phase	136	3.98	1.16	
	Relapse	69	3.84	1.58	
	Remission	64	3.88	1.60	

* Statistically significant difference at *p* < 0.05 confidence interval.

**Figure 1:**
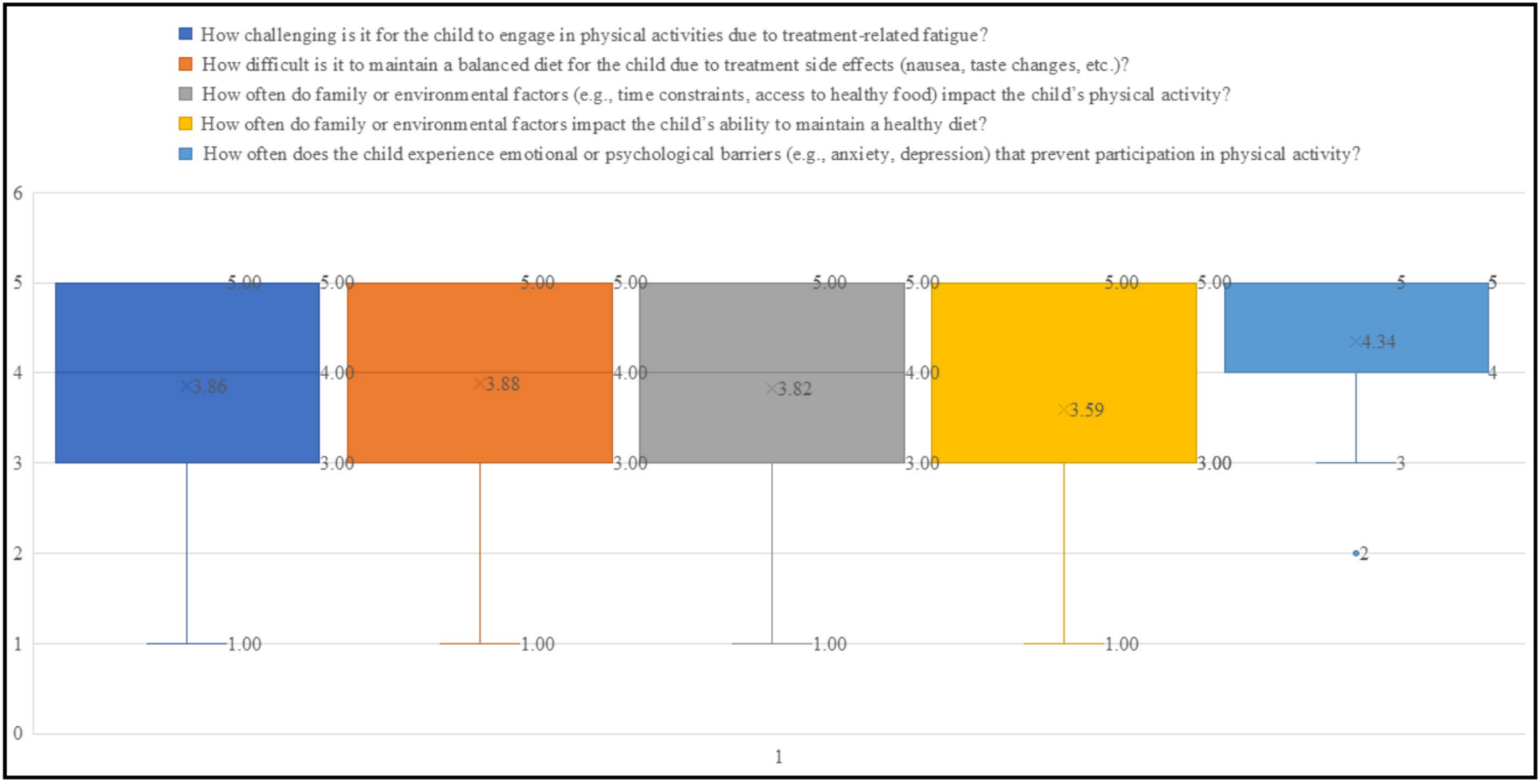
Box and Whisker plots for barriers to physical activity and nutrition.

The findings in Figure 1 highlights key barriers to physical activity and nutrition in children undergoing leukemia treatment. Psychological barriers such as anxiety and depression present the most significant challenge, with the highest mean score (4.34) and a median of 5, indicating that most caregivers perceive these factors as major obstacles to physical activity. Treatment-related fatigue is another substantial barrier, with a median of 5 and a mean of 3.86, reflecting its widespread impact on children’s ability to engage in exercise. Difficulties in maintaining a balanced diet due to treatment side effects, such as nausea and taste changes, also emerge as a challenge, with a median of 4 and a mean of 3.88. Family and environmental constraints, including access to healthy food and time limitations, influence both physical activity (mean 3.82) and dietary habits (mean 3.59), though diet appears to be slightly less affected. The wide range of responses (1–5) across all factors suggests varied caregiver experiences, emphasizing the need for personalized interventions, including psychological support, dietary guidance, and structured physical activity programs to mitigate these barriers effectively.

## Discussion

This study provides valuable insights into the physical activity levels and nutritional status of pediatric leukemia patients, highlighting significant variations based on gender, leukemia type, age, and treatment stage. These findings align with existing literature and provide further evidence of the challenges faced by children undergoing treatment.

A key finding was that 60% of participants failed to meet recommended physical activity levels, with children in the remission phase exhibiting significantly lower engagement in physical activities compared to those in the initial treatment or maintenance phases. This result aligns with previous studies (36–38), which found that fatigue and prolonged inactivity due to intensive treatment can contribute to lower physical activity levels. Additionally, the highest energy levels were reported in children in the maintenance phase, likely due to reduced treatment-related side effects, as supported by prior research (5–8). The significant variation in physical activity levels across treatment stages suggests that tailored exercise programs may be beneficial at different points in the treatment journey.

Gender differences were also observed, particularly in diet quality, where males had significantly better nutrient-rich diets compared to females. This finding mirrors previous research (21), which reported similar disparities in pediatric cancer patients’ nutritional intake. The reasons behind these differences may be multifaceted, potentially involving cultural factors, food preferences, or caregiver feeding behaviors. Future research should explore whether targeted nutritional interventions can address these disparities effectively.

Age-related differences were notable in motivation to participate in physical activities, with children aged 8–9 years reporting higher motivation compared to younger or older age groups. This supports earlier studies (39–41) suggesting that younger children may have developmental challenges that affect physical activity, while older children may experience psychological barriers such as anxiety and depression, limiting their engagement. These findings highlight the importance of age-specific interventions to encourage physical activity throughout treatment.

Furthermore, variations in physical activity and nutritional status based on leukemia type were observed. Children with ALL had significantly higher engagement in physical activities compared to those with MPAL, with a similar trend observed in energy levels. Prior research (42–44) suggests that different leukemia types and treatment protocols can impact physical health differently, which may explain these variations. Additionally, children with CLL and CML reported better-quality diets compared to those with MPAL, further emphasizing the need for individualized dietary strategies based on leukemia type.

One of the most critical findings was the impact of treatment phase on both physical activity and nutrition. Children in remission reported the lowest adherence to a regular eating schedule, which could be attributed to long-term side effects of treatment, as previously noted in studies (9–12). The significant decline in both physical activity and dietary adherence during remission underscores the need for continued support even after active treatment ends. Ensuring that children maintain healthy habits beyond intensive treatment could improve long-term health outcomes.

Overall, these findings suggest that physical activity and nutrition cannot be addressed in isolation, as they are interdependent and influenced by multiple factors, including treatment stage, gender, and leukemia type. This study reinforces the importance of personalized, multidisciplinary interventions, incorporating tailored exercise programs, gender-sensitive nutritional plans, and psychological support to optimize health outcomes in pediatric leukemia patients (29). Future research should focus on developing integrated care models that address these interrelated factors and evaluating their effectiveness in improving quality of life during and after leukemia treatment.

### Implications

Theoretically, this study contributes to the understanding of the complex relationships between physical activity, nutritional status, and treatment stages in pediatric leukemia patients. It highlights how different leukemia types, treatment phases, and gender impact physical health and nutrition, reinforcing the need for an integrative approach to pediatric cancer care. The findings emphasize that physical activity and nutrition cannot be addressed in isolation but must be considered together to optimize health outcomes during treatment. Practically, the results underscore the importance of personalized care interventions. Healthcare providers should develop tailored physical activity programs and individualized nutritional plans that cater to the specific needs of patients based on their treatment stage, leukemia type, and gender. Furthermore, the study suggests that addressing psychological barriers, such as anxiety and depression, is critical for enhancing both physical activity and nutritional intake. These practical strategies could improve the quality of life and treatment outcomes for pediatric leukemia patients, providing a more holistic approach to managing cancer care.

### Limitations

This study has several limitations that should be acknowledged. First, the use of self-reported data from caregivers may introduce bias, as responses might not fully capture the child’s actual physical activity levels or dietary habits, potentially leading to inaccuracies. Additionally, the cross-sectional design provides a snapshot of physical activity and nutritional status at one point in time, limiting the ability to assess long-term trends or changes throughout the treatment journey. The sample, while diverse, is geographically restricted to Saudi Arabia, which may limit the generalizability of the findings to other regions with different healthcare systems and cultural contexts. Furthermore, the study did not account for other factors such as socioeconomic status, which could influence access to nutritional resources or opportunities for physical activity. Future research could address these limitations by using objective measures of physical activity, adopting a longitudinal design, and expanding the sample to include diverse populations across multiple regions.

## Conclusion

This study provides important insights into the physical activity levels and nutritional status of pediatric leukemia patients, highlighting significant variations based on gender, leukemia type, age, and treatment stage. The findings demonstrate that treatment-related side effects such as fatigue, loss of appetite, and psychological distress significantly impact both physical activity and dietary habits, contributing to challenges in maintaining health during leukemia treatment. Gender differences in diet quality and age-related variations in motivation for physical activity further emphasize the need for personalized approaches to care. The study underscores the importance of integrating physical activity, nutrition, and psychological support into pediatric leukemia treatment plans to optimize outcomes and improve quality of life. Addressing these factors holistically, with tailored interventions based on individual patient needs, is critical for enhancing the overall well-being and recovery of children undergoing leukemia treatment. Future research should focus on exploring the long-term effects of physical activity and nutrition interventions and expanding this approach to diverse populations.

## Data Availability

The raw data supporting the conclusions of this article will be made available by the authors, without undue reservation.

## References

[1] Hunger SP Teachey DT Grupp S Aplenc R. Childhood leukemia. Abeloff's Clin Oncol. (2020) 2020:1748–1764.e4. doi: 10.1016/b978-0-323-47674-4.00093-1

[2] Mohammadian-Hafshejani A Farber IM Kheiri S. Global incidence and mortality of childhood leukemia and its relationship with the human development index. PLoS One. (2024) 19:e0304354. doi: 10.1371/journal.pone.0304354, PMID: 38954710 PMC11218982

[3] Madhusoodhan PP Carroll WL Bhatla T. Progress and prospects in pediatric leukemia. Curr Probl Pediatr Adolesc Health Care. (2016) 46:229–41. doi: 10.1016/j.cppeds.2016.04.003, PMID: 27283082

[4] Inaba H Pui C-H. Advances in the diagnosis and treatment of pediatric acute lymphoblastic leukemia. J Clin Med. (2021) 10:1926. doi: 10.3390/jcm10091926, PMID: 33946897 PMC8124693

[5] Manchola-González JD Bagur-Calafat C Girabent-Farrés M Serra-Grima JR Pérez RÁ Garnacho-Castaño MV. Effects of a home-exercise programme in childhood survivors of acute lymphoblastic leukaemia on physical fitness and physical functioning: results of a randomised clinical trial. Support Care Cancer. (2019) 28:3171–8. doi: 10.1007/s00520-019-05131-2, PMID: 31707503

[6] Barr RD Stevens MCG. The influence of nutrition on clinical outcomes in children with cancer. Pediatr Blood Cancer. (2020) 67:e28117. doi: 10.1002/pbc.28117, PMID: 32134218

[7] Rock CL Thomson CA Sullivan KR Howe CL Kushi LH Caan BJ. American Cancer Society nutrition and physical activity guideline for cancer survivors. CA Cancer J Clin. (2022) 72:230–62. doi: 10.3322/caac.21719, PMID: 35294043

[8] Wang S Maxwell CA Akella NM. Diet as a potential moderator for genome stability and immune response in pediatric leukemia. Cancers. (2021) 13:413. doi: 10.3390/cancers13030413, PMID: 33499176 PMC7865408

[9] Al-Mahayri ZN AlAhmad MM Ali BR. Long-term effects of pediatric acute lymphoblastic leukemia chemotherapy: can recent findings inform old strategies? Front Oncol. (2021) 11:710163. doi: 10.3389/fonc.2021.710163, PMID: 34722258 PMC8554193

[10] Hayashi H Makimoto A Yuza Y. Treatment of pediatric acute lymphoblastic leukemia: a historical perspective. Cancer. (2024) 16:723. doi: 10.3390/cancers16040723, PMID: 38398113 PMC10887299

[11] Pedretti L Massa S Leardini D Muratore E Rahman S Pession A. Role of nutrition in pediatric patients with Cancer. Nutrients. (2023) 15:710. doi: 10.3390/nu15030710, PMID: 36771416 PMC9920596

[12] Diakatou V Vassilakou T. Nutritional status of pediatric Cancer patients at diagnosis and correlations with treatment, clinical outcome and the long-term growth and health of survivors. Children. (2020) 7:218. doi: 10.3390/children7110218, PMID: 33171756 PMC7694979

[13] Rapti C Dinas PC Chryssanthopoulos C Mila A Philippou A. Effects of exercise and physical activity levels on childhood Cancer: an umbrella review. Healthcare. (2023) 11:820. doi: 10.3390/healthcare11060820, PMID: 36981477 PMC10048410

[14] Friedenreich CM Stone CR Cheung WY Hayes SC. Physical activity and mortality in Cancer survivors: a systematic review and Meta-analysis. JNCI Cancer Spectr. (2019) 4:pkz080. doi: 10.1093/jncics/pkz080, PMID: 32337494 PMC7050161

[15] Simioni C Zauli G Martelli AM Vitale M Ultimo S Milani D. Physical training interventions for children and teenagers affected by acute lymphoblastic leukemia and related treatment impairments. Oncotarget. (2018) 9:17199–209. doi: 10.18632/oncotarget.24762, PMID: 29682216 PMC5908317

[16] Alias H Mohd Nazi NA Lau Sie Chong D. Participation in physical activity and physical education in school among children with acute lymphoblastic leukemia after intensive chemotherapy. Front Pediatr. (2019) 7:73. doi: 10.3389/fped.2019.00073, PMID: 30937299 PMC6431648

[17] Milliron BJ Packel L Dychtwald D Klobodu C Pontiggia L Ogbogu O. When eating becomes torturous: understanding nutrition-related Cancer treatment side effects among individuals with cancer and their caregivers. Nutrients. (2022) 14:356. doi: 10.3390/nu14020356, PMID: 35057538 PMC8781744

[18] Brinksma A Sulkers E IJpma I JGM B WJE T. Eating and feeding problems in children with cancer: prevalence, related factors, and consequences. Clin Nutr. (2020) 39:3072–9. doi: 10.1016/j.clnu.2020.01.012, PMID: 32057537

[19] Supportive PD Board PC (2023). Nausea and vomiting related to cancer treatment (PDQ®). Cancer.gov. Available online at: https://www.cancer.gov/about-cancer/treatment/side-effects/nausea/nausea-hp-pdq (Accessed August 8, 2024).

[20] Sonneborn-Papakostopoulos M Dubois C Mathies V Heß M Erickson N Ernst T. Quality of life, symptoms and dietary habits in oncology outpatients with malnutrition: a cross-sectional study. Med Oncol. (2021) 38:20. doi: 10.1007/s12032-021-01460-7, PMID: 33543336 PMC7862192

[21] Adamovich T Watson R Murdoch S Giovino L Kulkarni S Luchak M. Barriers and facilitators to physical activity participation for child, adolescent, and young adult cancer survivors: a systematic review. J Cancer Surviv. (2022) 18:245–62. doi: 10.1007/s11764-022-01217-9, PMID: 35665472

[22] Karalexi MA Markozannes G Tagkas CF Katsimpris A Tseretopoulou X Tsilidis KK. Nutritional status at diagnosis as predictor of survival from childhood Cancer: a review of the literature. Diagnostics. (2022) 12:2357. doi: 10.3390/diagnostics12102357, PMID: 36292046 PMC9600212

[23] Kandemir I Anak S Karaman S Yaman A Varkal MA Devecioglu O. Nutritional status of pediatric patients with acute lymphoblastic leukemia under chemotherapy: a pilot longitudinal study. J Pediatr Hematol Oncol. (2023) 45:235–40. doi: 10.1097/MPH.0000000000002685, PMID: 37278580

[24] Joffe L Ladas EJ. Nutrition during childhood cancer treatment: current understanding and a path for future research. Lancet Child Adolesc Health. (2020) 4:465–75. doi: 10.1016/S2352-4642(19)30407-9, PMID: 32061318

[25] Tripodi SI Bergami E Panigari A Caissutti V Brovia C de Cicco M. The role of nutrition in children with cancer. Tumori. (2023) 109:19–27. doi: 10.1177/03008916221084740, PMID: 35722985 PMC9896537

[26] West SL Banks L Schneiderman JE Caterini JE Stephens S White G. Physical activity for children with chronic disease; a narrative review and practical applications. BMC Pediatr. (2019) 19:12. doi: 10.1186/s12887-018-1377-3, PMID: 30621667 PMC6325687

[27] Oberoi S Robinson PD Cataudella D Culos-Reed SN Davis H Duong N. Physical activity reduces fatigue in patients with cancer and hematopoietic stem cell transplant recipients: a systematic review and meta-analysis of randomized trials. Crit Rev Oncol Hematol. (2017) 122:52–9. doi: 10.1016/j.critrevonc.2017.12.011, PMID: 29458789

[28] Brinksma A Huizinga G Sulkers E Kamps W Roodbol P Tissing W. Malnutrition in childhood cancer patients: a review on its prevalence and possible causes. Crit Rev Oncol Hematol. (2012) 83:249–75. doi: 10.1016/j.critrevonc.2011.12.003, PMID: 22264939

[29] Ward EJ Henry LM Friend AJ Wilkins S Phillips RS. Nutritional support in children and young people with cancer undergoing chemotherapy. Cochrane Database Syst Rev. (2015) 2015:CD003298. doi: 10.1002/14651858.CD003298.pub3, PMID: 26301790 PMC8752126

[30] Ponzo V Goitre I Favaro E Merlo FD Mancino MV Riso S. Is ChatGPT an effective tool for providing dietary advice? Nutrients. (2024) 16:469. doi: 10.3390/nu16040469, PMID: 38398794 PMC10892804

[31] Bermingham KM Linenberg I Polidori L Asnicar F Arrè A Wolf J. Effects of a personalized nutrition program on cardiometabolic health: a randomized controlled trial. Nat Med. (2024) 30:1888–97. doi: 10.1038/s41591-024-02951-6, PMID: 38714898 PMC11271409

[32] Egondi T Kyobutungi C Ng N Muindi K Oti S Vijver SV. Community perceptions of air pollution and related health risks in Nairobi slums. Int J Environ Res Public Health. (2013) 10:4851–68. doi: 10.3390/ijerph10104851, PMID: 24157509 PMC3823347

[33] Ahmad H Halim H. Determining sample size for research activities. Selangor Bus Rev. (2017) 2:20–4.

[34] Cha E Kim KH Erlen JA. Translation of scales in cross-cultural research: issues and techniques. J Adv Nurs. (2007) 58:386–95. doi: 10.1111/j.1365-2648.2007.04242.x, PMID: 17442038

[35] Taber KS. The use of Cronbach’s alpha when developing and reporting research instruments in science education. Res Sci Educ. (2018) 48:1273–96. doi: 10.1007/s11165-016-9602-2, PMID: 40078838

[36] Blaney J Lowe-Strong A Rankin J Campbell A Allen J Gracey J. The Cancer rehabilitation journey: barriers to and facilitators of exercise among patients with Cancer-related fatigue. Phys Ther. (2010) 90:1135–47. doi: 10.2522/ptj.20090278, PMID: 20558566

[37] Götte M Kesting S Winter C Rosenbaum D Boos J. Experience of barriers and motivations for physical activities and exercise during treatment of pediatric patients with cancer. Pediatr Blood Cancer. (2014) 61:1632–7. doi: 10.1002/pbc.25071, PMID: 24753116

[38] Guimarães JAC Guerra PH Ueno DT Spósito LAC Sebastião E Nakamura PM. Barriers to physical activity among cancer pediatric cancer patients and survivors: a scoping review. Motriz Revista De Educação Física. (2021) 28:28. doi: 10.1590/s1980-657420220005621

[39] Mihic-Góngora L Jimenez-Fonseca P Coca-Membribes S Cruz-Castellanos P Galán-Moral R Asensio-Martínez E. Physical activity in patients with advanced Cancer: sociodemographic, clinical, and psychological correlates. Brain Sci. (2024) 14:573. doi: 10.3390/brainsci14060573, PMID: 38928572 PMC11201712

[40] Niedzwiedz CL Knifton L Robb KA Katikireddi SV Smith DJ. Depression and anxiety among people living with and beyond cancer: a growing clinical and research priority. BMC Cancer. (2019) 19:943. doi: 10.1186/s12885-019-6181-4, PMID: 31604468 PMC6788022

[41] Gilliam MB Schwebel DC. Physical activity in child and adolescent Cancer survivors: a review. Health Psychol Rev. (2013) 7:92–110. doi: 10.1080/17437199.2011.603641, PMID: 25484907 PMC4257474

[42] Brown JC Winters-Stone K Lee A Schmitz KH. Cancer, physical activity, and exercise. Compr Physiol. (2012) 2:2775–809. doi: 10.1002/cphy.c120005, PMID: 23720265 PMC4122430

[43] Bryant AL Deal AM Battaglini CL Phillips B Pergolotti M Coffman E. The effects of exercise on patient-reported outcomes and performance-based physical function in adults with acute leukemia undergoing induction therapy: exercise and quality of life in acute leukemia (EQUAL). Integr Cancer Ther. (2018) 17:263–70. doi: 10.1177/1534735417699881, PMID: 28627275 PMC6041904

[44] Tan SY Poh BK Chong HX Ismail MN Rahman J Zarina AL. Physical activity of pediatric patients with acute leukemia undergoing induction or consolidation chemotherapy. Leuk Res. (2012) 37:14–20. doi: 10.1016/j.leukres.2012.09.005, PMID: 23099236

